# Adenosine A_2A_ receptors regulate D_2_-type medium spiny neurons in the nucleus accumbens to mediate pain and depression comorbidity

**DOI:** 10.3389/fphar.2026.1759544

**Published:** 2026-02-23

**Authors:** Dongyu Zhou, Xiaona Yang, Lingzhen Song, Mengyu Wu, Hongxing Zhang, Jun-Li Cao, Su Min

**Affiliations:** 1 Department of Anesthesiology, the First Affiliated Hospital of Chongqing Medical University, Chongqing, China; 2 Department of Pain Management, Lianyungang Clinical College of Nanjing Medical University, Lianyungang, China; 3 Jiangsu Province Key Laboratory of Anesthesiology, Xuzhou Medical University, Xuzhou, China; 4 Jiangsu Province Key Laboratory of Anesthesia and Analgesia Application Technology, Xuzhou Medical University, Xuzhou, China; 5 NMPA Key Laboratory for Research and Evaluation of Narcotic and Psychotropic Drugs, Xuzhou Medical University, Xuzhou, China; 6 Nanjing Medical University, Nanjing, China

**Keywords:** A2A receptors, comorbidity, D2-MSNs, depression, NAc shell, pain

## Abstract

Persistent pathological pain often induces comorbid depressive-like symptoms, yet the underlying neuronal and molecular mechanisms remain unclear. The nucleus accumbens shell (NAcS) has been implicated in mediating pain sensation and emotional disorders, including depression. However, how it regulates pain-depression comorbidity (PDC) is not well-known. In the present study, we demonstrated that D_2_-type medium spiny neurons (D_2_-MSNs) in the NAcS bidirectionally modulated pain and its comorbid behavioral despair measured in the forced swimming test (FST), an effect possibly involving adenosine A_2A_ receptors (A_2A_Rs). Specifically, acute chemogenetic activation of NAcS D_2_-MSNs induced thermal/mechanical pain in naïve mice but did not affect despair-like behavior in the FST, a phenomenon that occurred after repeated activation of these neurons. Conversely, chemogenetic inhibition of NAcS D_2_-MSNs alleviated spared nerve injury (SNI) induced neuropathic pain and its comorbid behavioral despair. Our immunofluorescent staining revealed a relatively enriched expression of A_2A_Rs in NAcS D_2_-MSNs. *Ex vivo* electrophysiological recordings revealed that activating and inhibiting A_2A_Rs increased and decreased the neuronal excitability of NAcS D_2_-MSNs. Consistently, local infusion of the agonist and antagonist of A_2A_Rs into the NAcS bidirectionally modulated pain and despair-like behaviors in both naïve and SNI mice. Together, these findings demonstrate the functional role of NAcS D_2_-MSNs in mediating PDC, which was possibly modulated by local A_2A_Rs, thus providing a potential therapeutic target for PDC.

## Introduction

Chronic pain affects more than 30% of people worldwide (Cohen et al., 2021), and approximately 30%–40% of those individuals also develop clinically significant depressive symptoms (Aaron et al., 2025). This pain-depression comorbidity (PDC) not only exacerbates disability and diminishes quality of life but also imposes a substantial and escalating socioeconomic burden. Despite extensive clinical attention, PDC remains challenging to treat, as current pharmacological treatments provide only limited clinical benefit. Elucidating the neural mechanisms underlying the onset and progression of PDC remains a major challenge.

The nucleus accumbens shell (NAcS) is a critical hub in the mesocorticolimbic reward pathway, receiving ascending nociceptive inputs and integrating limbic and cortical top-down control (Castro et al., 2019; Mitrano et al., 2012; Xie et al., 2014). The NAcS is increasingly recognized as a key node involved in pain processing and psychiatric disorders, particularly depression. However, while the involvement of the NAcS in pain-related affective behavior has been well established, the circuit- and cell-type-specific mechanisms through which the NAcS integrates nociceptive and affective signals under PDC remain incompletely understood. In the NAcS, medium spiny neurons (MSNs) are classified based on dopamine receptor expression into D_1_-MSNs and D_2_-MSNs (Le Moine and Bloch, 1996; Lu et al., 1998; Ikemoto et al., 1997). Studies targeting the NAcS have implicated D_2_-MSNs in neuropathic pain and depression-like behaviors (Ren et al., 2016; Wu et al., 2018). However, how the activity of D_2_-MSNs is modulated at the receptor and intracellular signaling levels under chronic pain-associated affective states remains poorly understood. Identifying cell-type-specific molecular mechanisms enriched in D_2_-MSNs that regulate their excitability may therefore reveal novel therapeutic targets for PDC.

Adenosine A_2A_ receptors (A_2A_Rs) have emerged as potential therapeutic targets for both pain and affective disorders, such as depression and anxiety (Pasquini et al., 2022; Sardi et al., 2018; Kaster et al., 2015). In a mouse model of trigeminal neuralgia, glia-driven increases in extracellular adenosine in the ventral hippocampal CA1 region promote pyramidal neuron hyperexcitability and consequent anxiodepressive-like behaviors, supporting A_2A_R recruitment in pain-depression coupling (Lv et al., 2024). Notably, the influence of A_2A_R signaling on nociception is highly region-dependent. Microinjection of A_2A_R agonists into the paraventricular thalamus (PVT) induced hyperalgesia, whereas antagonists mitigated Complete Freund’s Adjuvant (CFA)-induced inflammatory pain (Cao et al., 2024). In contrast, spinal A_2A_R activation has been shown to exert protective and antinociceptive effects in spinal cord injury models, accompanied by suppression of glial activation and pro-inflammatory signaling (Irrera et al., 2018). These findings underscore the anatomically specific and context-dependent functions of A_2A_R signaling in pain modulation. In the NAcS, A_2A_Rs are abundantly expressed (Svenningsson et al., 1997). A_2A_R signaling has been directly implicated in nociceptive modulation (Sardi et al., 2018). In a rat model of rapid eye movement sleep deprivation (REM-SD)-induced hyperalgesia, intra-NAc activation of A_2A_Rs exacerbated pain hypersensitivity, whereas pharmacological blockade of A_2A_Rs alleviated hyperalgesia (Sardi et al., 2018). Moreover, A_2A_R signaling in the NAcS is also closely associated with stress-related affective vulnerability. In a mouse chronic social defeat stress (CSDS) model, lower A_2A_ expression in the NAc was observed in stress-susceptible mice and correlated with increased anxiety-like behavior and anhedonia, whereas relatively higher A_2A_ expression is associated with stress resilience (Bevilacqua et al., 2026). Together, these findings suggest that NAcS A_2A_R signaling has emerged as a critical therapeutic target for both pain and affective disorders. However, these studies primarily examined nociceptive or affective phenotypes in isolation, leaving unresolved how NAcS A_2A_R signaling integrates pain and depressive components under PDC.

At the cellular level, A_2A_Rs and D_2_Rs are G protein-coupled receptors that exert well-established but opposing canonical actions in striatal medium spiny neurons (Canals et al., 2003; Kamiya et al., 2003). Evidence indicates that A_2A_Rs are preferentially expressed in striatal D_2_-MSNs (Fink et al., 1992; Rosin et al., 2003), where their activation recruits cAMP/PKA signaling to enhance the intrinsic excitability and firing activity of D_2_-MSNs (Wang and Zhou, 2019). In contrast, D_2_Rs suppress cAMP signaling, generally decreasing neuronal excitability and functionally dampening NMDAR-dependent excitatory responses (Azdad et al., 2009). Together, these opposing receptor mechanisms suggest that A_2A_R signaling is a key molecular determinant of D_2_-MSNs’ excitability. Although the role of A_2A_Rs in regulating D_2_-MSNs excitability under basal conditions has been well established, the prevalence and functional relevance of A_2A_Rs and D_2_-MSNs under chronic neuropathic pain conditions remain unclear. Whether and how these A_2A_R-dependent mechanisms contribute to PDC by regulating the intrinsic excitability of D_2_-MSNs remains largely unknown.

In this study, we employed selective pharmacological manipulation of A_2A_Rs combined with chemogenetics, immunofluorescent co-labeling, and whole-cell patch-clamp recordings in a mouse model of SNI-induced PDC. We specifically investigated how A_2A_R signaling in the NAcS modulates D_2_-MSNs’ intrinsic excitability under chronic pain conditions and how this modulation contributes to the behavioral phenotypes of PDC. Our findings identify NAcS A_2A_R signaling as a modulator of D_2_-MSNs function in pain-associated affective dysfunction and suggest this pathway as a candidate target for future therapeutic strategies.

## Materials and methods

### Animal and housing

All experimental procedures were reviewed and approved by the Animal Care and Use Committee of Xuzhou Medical University (Approval No. 202207S034) and conducted in accordance with the National Institutes of Health Guidelines for the Care and Use of Laboratory Animals and the ethical standards of the International Association for the Study of Pain. Mice were housed in groups of up to five per cage under a 12 h light/dark cycle (lights on from 08:00 to 20:00), with food and water available *ad libitum*. Ambient temperature was maintained at 23 °C ± 2 °C with relative humidity at 55%–60%. Only male C57BL/6J mice aged 8–14 weeks (20–30 g) without signs of illness, injury, or abnormal grooming/locomotion were used for the experiments. Food and water were withheld during the modeling and testing sessions (1–2 h). All behavioral tests were conducted during the light phase (09:00–16:00), and experimenters were blinded to the treatment conditions throughout testing. All efforts were made to minimize animal suffering and to reduce the number of animals used.

### Chemogenetic manipulation and viral vectors

All AAV vectors were purchased from BrainVTA Technology (Wuhan, China): rAAV-D_2_-mCherry-WPREs-bGH (AAV2/9, BrainVTA Technology, PT-0367), rAAV-D_2_-hM3D(Gq)-mCherry-WPREs (AAV2/9, BrainVTA Technology, PT-8500), and rAAV-D_2_-hM4D(Gi)-mCherry-WPRE-hGH pA (AAV2/9, BrainVTA Technology, PT-8119). The D_2_-promoter viral approach was supported by published co-labeling validation in the NAcS, confirming the virus’s high specificity for targeting D_2_R-positive neurons (Xia et al., 2025). Viruses were bilaterally injected into the NAcS (AP = +1.65 mm, ML = ±0.55 mm, DV = −4.65 mm) at a volume of 200 nL per side using a microsyringe pump. After injection, the needle was left in place for 10 min to minimize reflux. Experiments were performed 3 weeks after viral injection to allow for stable transgene expression. For chemogenetic experiments, control mice received rAAV-D_2_-mCherry-WPREs-bGH to control for viral infection and fluorophore expression without DREADD activation. Clozapine-N-oxide (CNO; MedChemExpress, 34233–69–7) was dissolved in sterile saline containing 0.1% DMSO and administered intraperitoneally (3 mg/kg) 30 min before behavioral testing. To control for potential off-target effects of CNO and its back-metabolism to clozapine, mice in both DREADD-expressing and control-virus groups received the same dose and injection volume of CNO. Vehicle controls (when applicable) received an equivalent volume of saline containing 0.1% DMSO.

### Stereotactic surgeries

Mice were anesthetized with 1% pentobarbital sodium (40 mg/kg, i.p.) via intraperitoneal injection and positioned on a small animal stereotaxic frame (RWD Life Technology Co., Ltd., Shenzhen, China). Ophthalmic ointment was applied to the mice’s eyes throughout the surgery to maintain moisture. A 1 cm scalp incision was made to expose the skull, and the skull was adjusted to align the bregma and lambda horizontally. Small holes were drilled above the target brain region using a dental drill, and bilateral cannula (RWD Life Technology Co., Ltd.) were fixed to the skull with acrylic cement and inserted into the NAcS (AP = +1.65 mm, ML = ±0.55 mm, DV = −4.65 mm). Stainless-steel obturators were inserted into each cannula to prevent occlusion. After the surgery, the mice were placed in a cage with a heating pad to maintain body temperature and returned to their home cage once they were fully awake. Mice were allowed to recover for at least 1 week before behavioral tests. The A_2A_R agonist CGS 21680 (MedChemExpress, HY-13201A) and antagonist SCH 58261 (MedChemExpress, HY-19533) were initially dissolved in 100% dimethyl sulfoxide (DMSO) to prepare 10 mM stock solutions and stored at −20 °C. On the day of the experiment, stock solutions were diluted with sterile saline to the desired working concentrations, with the final DMSO concentration kept below 0.1%. The doses used were selected based on our previous studies and preliminary experiments. Control mice received equivalent volumes of saline vehicle. Drugs or vehicle solution (saline containing the same concentration of DMSO) were bilaterally microinjected into the NAcS at a volume of 0.5 μL per side and a rate of 0.2 μL/min. The injection cannula was left in place for an additional 5 min to prevent backflow before removal. After the microinjection, the mice were returned to their home cages for 30 min before behavioral tests.

### Spared nerve injury (SNI) model of neuropathic pain

The SNI surgery was performed in accordance with established protocols (Richner et al., 2011; Cichon et al., 2018). Mice were anaesthetized using sodium pentobarbital (40 mg/kg, i.p.). Clean skin on the lateral surface of the left thigh and then dissect the skin and muscles bluntly to reveal the three branches of the sciatic nerve: the common peroneal nerve, the tibial nerve, and the sural nerve. At the bifurcation of the sciatic nerve, the common peroneal and tibial nerve branches were tightly ligated with 6–0 non-absorbable sutures and transected distal to the ligation site, while the sural nerve was left intact. In the sham group, nerves were exposed and dissected without any sectioning. After the skin was closed, the surgical site was disinfected with povidone-iodine.

## Von Frey test and 50% paw withdrawal thresholds (50% PWTs)

Mechanical thresholds were determined using the up-down (Dixon) method (Chaplan et al., 1994; Hirsh et al., 2007). The PWTs were assessed using von Frey filaments (Stoelting, Kiel, WI, United States). Stimuli of 0.008–6 g were applied to the lateral third of the plantar surface of the left hind paw. A positive response was defined as an abrupt paw withdrawal, twitch, or licking, whereas the absence of these behaviors was considered a negative response. Mechanical thresholds were determined using the up-down (Dixon) method. Mice were acclimated for 1 h on a metal mesh platform inside a transparent acrylic chamber (8 × 8 × 5.5 cm) in a quiet, temperature-controlled room. Testing began with a 0.16 g filament. If no positive response occurred, the next higher stimulus was applied; if a positive response was observed, the next lower stimulus was used. This procedure continued until the first alternation between a positive and a negative response was obtained. Subsequently, four additional measurements were obtained, with at least 30 s between each stimulus. Response patterns were recorded in a table, with “X” denoting a positive response and “O” indicating a negative response. The 50% PWTs were calculated using the following formula: 50% PWTs = 10^[log(Xf) + kδ], where Xf is the final filament value (in log units), k is the coefficient determined by the response pattern, and δ represents the mean interstimulus interval (log units; δ = 0.410723). All behavioral tests were performed by an experimenter blinded to the treatment conditions.

### Hargreaves test and paw withdrawal latency (PWL)

Thermal nociception was assessed using the Hargreaves method (Hargreaves et al., 1988). Mice were individually placed on an elevated glass platform within a transparent acrylic container (8 cm long × 8 cm wide × 5.5 cm high) for 1 h in a temperature-controlled, quiet room. A focused beam of light was directed onto the plantar surface of the left hindlimb. The time from the onset of the thermal stimulus to hindlimb withdrawal is recorded as the paw withdrawal latency (PWL). This latency reflects the mouse’s pain threshold. The thermal stimulus duration was limited to 20 s to prevent tissue damage. Testing was repeated 5 times with a 5 min interval between trials, and the average time was recorded as the PWL. All tests were conducted blind.

### Forced swimming test (FST)

The forced swimming test was performed as previously described (Porsolt et al., 1977; Cryan et al., 2002). The mouse was individually placed in a transparent acrylic cylinder (25 cm in height, 12.5 cm in diameter) filled with water to a depth of 15 cm, maintained at 25 °C ± 1 °C. After a 1 min habituation period in the water, immobility time was recorded for the following 5 min.

### Open field test (OFT)

The open field test was performed as previously described (Seibenhener and Wooten, 2015). The mouse was placed individually in a transparent, white acrylic arena (45 cm × 45 cm). After a 1 min habituation period, the mouse’s movement was recorded for the following 10 min. Total distance traveled, velocity, and the amount of time spent in the center versus the periphery of the arena were measured using a video tracking system.

### Immunohistochemistry

Immunohistochemistry was performed as previously described (Cao et al., 2024). Mice were anesthetized with 1% sodium pentobarbital (40 mg/kg, i.p.) and secured in a supine position on a perfusion table. A midline incision was made along the lower costal margin to expose the thoracic cavity. The diaphragm and both sides of the sternum were sequentially incised to open the thoracic cavity and expose the heart. The pericardium was removed with forceps. A perfusion needle was inserted along the longitudinal axis of the heart into the left ventricle and secured in place. The right atrial appendage was incised, and mice were perfused with 20 mL of cold 0.01 M phosphate-buffered saline (PBS, pH 7.4), followed by 20 mL of PBS containing 4% paraformaldehyde (PFA). Successful perfusion was indicated by pallor of the extremities and whole-body convulsions. After perfusion, the head was severed using tissue scissors. The occipital, parietal, and frontal bones were removed sequentially with care to avoid damaging the brain. All twelve cranial nerves were transected using forceps. The intact brain was carefully extracted and post-fixed in 4% PFA at 4 °C for 12 h. The next day, the PFA solution was replaced with 30% sucrose for cryoprotection. Once the brain sank to the bottom of the sucrose solution, sectioning and staining were performed. The brain was embedded in tissue embedding medium on an ice-cold platform at −20 °C. After freezing, coronal sections (30 μm thick) were cut using a Leica cryostat and collected in PBS.

Sections were incubated overnight at 4 °C in primary antibody dilution buffer (PBST; PBS containing 0.1% Tween-20) containing one of the following primary antibodies: mouse anti-A2AR (1:100, Santa Cruz, sc-32261), rabbit anti-Iba1 (1:500, Wako, 016–20001), rabbit anti-GFAP (1:500, Proteintech, 16825-1-AP), rabbit anti-NeuN (1:500, Proteintech, 26975-1-AP), rabbit anti-DRD1 (1:100, HUABIO, HA751324), or rabbit anti-DRD2 (1:100, HUABIO, ET1703-45). Each primary antibody was applied to separate sections. The next day, sections were washed three times with PBST (5 min each) and then incubated with the corresponding secondary antibody diluted in PBST (anti-mouse Alexa 488, 1:500, Thermo Fisher Scientific, A28175; or anti-rabbit Alexa 594, 1:500, Thermo Fisher Scientific, A11012) for 2 h at room temperature in the dark. Sections were then washed three times with PBST (5 min each) to remove residual secondary antibody, mounted onto slides, and coverslipped with an anti-fade mounting medium. Slides were stored at −20 °C in the dark. Images were acquired using a Zeiss laser scanning confocal microscope (LSM880).

### Whole-cell patch-clamp recordings

Whole-cell patch-clamp recordings were performed in acute NAcS-containing brain slices as previously described (Zhao et al., 2024). Electrophysiological recordings were performed 6–10 weeks after SNI surgery, at the time when stable PDC was established. The mice were anesthetized with isoflurane, and brains were rapidly removed and immersed in an ice-cold sucrose-based cutting solution (0 °C–4 °C) for approximately 2 min. Brains were then sectioned into 300-μm-thick slices containing the NAcS using a vibratome (DTK-1000). The sucrose-based cutting solution contained (in mM): 254 sucrose, 3 KCl, 1.25 NaH_2_PO_4_, 10 D-glucose, 24 NaHCO_3_, 2 CaCl_2_, and 2 MgSO_4_ (pH 7.35, 295–305 mOsm). The brain slices were incubated in artificial cerebrospinal fluid (ACSF) equilibrated with 95% O_2_ and 5% CO_2_ and containing (in mM): 128 NaCl, 3 KCl, 1.25 NaH_2_PO_4_, 10 D-glucose, 24 NaHCO_3_, 2 CaCl_2_, and 2 MgSO_4_ (pH 7.35, 295–305 mOsm)] for 50 min, then equilibrated at room temperature for approximately 1 h before use. For recording, the brain slices were placed in the recording chamber and continuously perfused with ACSF equilibrated with 95% O_2_ and 5% CO_2_. Suitable cells were located under a microscope, and a glass pipette electrode (5–8 MΩ) was slowly approached to the cell surface. After achieving a gigaohm(GΩ) seal, membrane rupture was induced by suction to establish the whole-cell recording configuration. Patch pipettes were filled with an internal solution containing (in mM): 130 K-gluconate, 10 KCl, 10 HEPES, 0.5 EGTA, 2 MgCl_2_, 2 ATP-Na_2_, and 0.3 GTP-Na, pH 7.3, 285 mOsm. To minimize pseudoreplication, we followed standardized recording practices: (1) we recorded from 2-3 brain slices per animal; (2) we recorded no more than 3 cells per slice, with recorded cells separated by at least 100 μm.

### Statistics

Data normality was assessed using the *Shapiro–Wilk test*. Non-normally distributed data were analyzed with nonparametric methods. For normally distributed data with equal variances, two-group comparisons were performed using unpaired *two-tailed t tests*. For multiple groups, *one- or two-way ANOVA* was applied as appropriate, followed by *Tukey’s* post hoc test. When normality assumptions were not met, two-group comparisons used the *Mann–Whitney U test*; comparisons among more than two independent groups used the *Kruskal–Wallis test*; and repeated-measures analyses across related groups used the *Friedman test*, with *Dunn’s multiple comparisons test* for post hoc analysis. For electrophysiological experiments, data were analyzed at the level of individual cells (n), and the number of animals (N) from which cells were recorded is consistently reported throughout the manuscript. To address potential concerns about pseudoreplication arising from recording multiple cells from the same animal, we performed complementary analyses at the animal level for all key findings. Specifically, we calculated the mean value for all cells recorded from each animal and then performed statistical tests using these animal-level means. These animal-level analyses confirmed that all major findings remained statistically significant, demonstrating that our conclusions are robust regardless of whether cells or animals are treated as the unit of analysis. Data were presented as *mean ± SEM*. Statistical significance was set at *P* < 0.05 (*P < 0.05, **P < 0.01, and ***P < 0.001). Statistical analyses were conducted in *GraphPad Prism 8.0*.

## Results

### D_2_-MSNs exhibit increased intrinsic excitability in PDC mice

**FIGURE 1:**
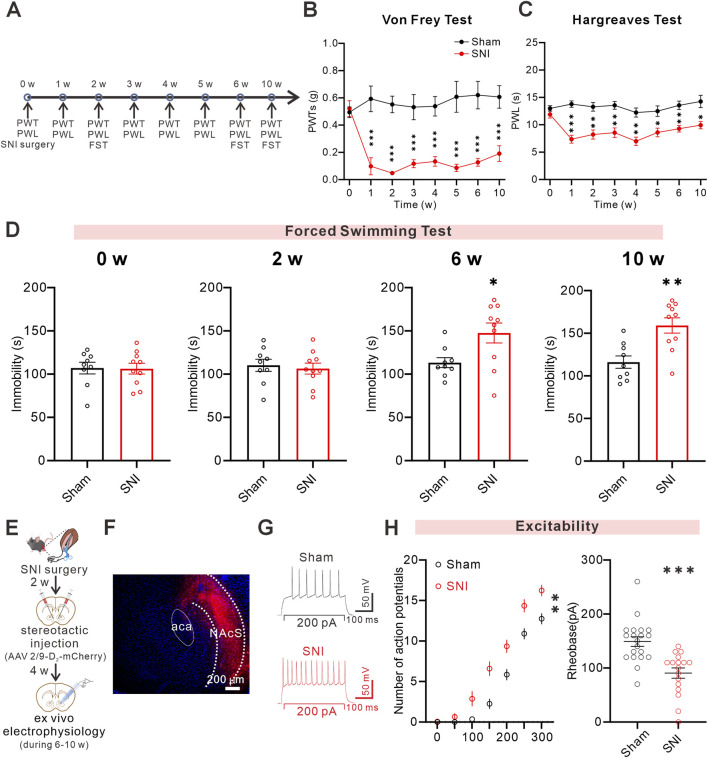
D_2_-MSNs showed increased excitability in pain-depression comorbidity mice **(A)** Timeline of the SNI surgery procedure and experimental protocol **(B,C)** 50%PWTs **(B)** and PWL **(C)** at different time points after SNI (Sham, n = 9 mice; SNI, n = 10 mice) **(D)** The immobility in the FST and at 0 W, 2 W, 6 W, and 10 W after SNI **(E)** Timeline of SNI surgery, stereotactic injection, and *ex vivo* electrophysiology at specified time points **(F)** Representative confocal images for virus expression in NAcS, scale bar = 200 µm **(G)** Sample of whole-cell recording of action potentials in the NAcS **(H)** (left) Quantitative data showing a significantly increased number of eAPs of mCherry-labeled neurons in SNI mice (right) The minimal voltage threshold to induce eAPs was lower in SNI mice (n = 20, 17 cells from 4, 4 mice, respectively). Data are represented as *mean ± s.e.m.*, *P < 0.05, **P < 0.01, ***P < 0.001.

Previous studies have shown that D_2_-MSNs play an essential regulatory role in pain and negative emotions (Ren et al., 2016; Wu et al., 2018). To explore how NAcS D_2_-MSNs respond under PDC, we established a neuropathic pain-related SNI mouse model by ligating and transecting the common peroneal and tibial nerves while the sural nerve was left intact (Figure 1A). As previously reported, SNI mice exhibited a sustained decrease in 50%PWTs and PWL (Li et al., 2021). Behavioral tests conducted at multiple time points revealed that, compared to Sham mice, SNI mice showed a significant decline in 50%PWTs (Figure 1B) and PWL (Figure 1C) that persisted for at least 10 weeks. In addition, at week 6 post-surgery, SNI mice displayed a significant increase in immobility time in the forced swimming test (FST), which persisted at least until the 10 th week (Figure 1D). These findings suggest that SNI-induced neuropathic pain induces stable comorbid behaviors, providing a reliable time frame for subsequent functional studies.

Next, we investigated alterations in the excitability of D_2_-MSNs in the NAcS under PDC. First, we injected AAV2/9-D_2_-mCherry into the NAcS to label D_2_-MSNs (Figures 1E,F). At 6–10 weeks post-SNI surgery, we performed whole-cell patch-clamp recordings in acutely prepared NAcS slices and quantified neuronal excitability by measuring depolarizing current-evoked action potentials (eAPs). (Figure 1E). Compared with Sham mice, SNI-PDC mice exhibited a significant increase in evoked action potential firing and a reduced action potential threshold (Figures 1G,H). These results collectively indicate that the intrinsic excitability of NAcS D_2_-MSNs is markedly upregulated in PDC mice.

### Chemogenetic manipulation of NAcS D_2_-MSNs activity bidirectionally regulates pain and despair-like behaviors

**FIGURE 2:**
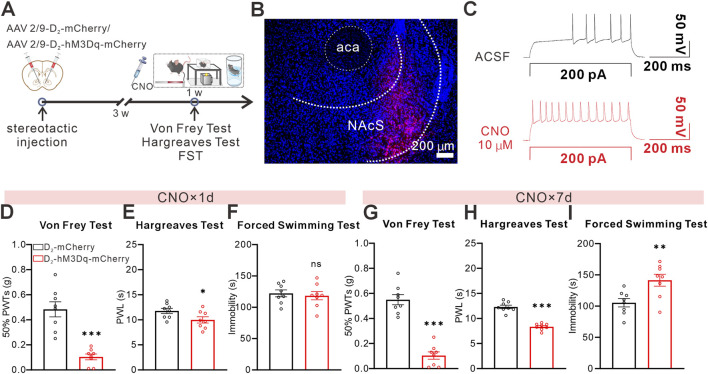
Chemogenetic activation of NAcS D_2_-MSNs induced pain and depression-like behaviors in naïve mice **(A)** Timeline of the stereotactic injection and behavioral tests **(B)** Representative confocal images for virus expression in NAcS, scale bar = 200 µm **(C)** Sample trace of whole-cell recording showing the increased excitatory effect of CNO on an hM3Dq-mCherry-expressing NAcS neuron **(D–F)** 50%PWTs **(D)**, PWL **(E)**, and immobile time in the FST **(F)** 30 min after a single CNO administration (n = 8 mice/group) **(G–I)** 50%PWTs **(G)**, PWL **(H)**, and immobile time in the FST **(I)** 30 min after 7 days repeated CNO administration (n = 8 mice/group). Data are represented as *mean ± s.e.m.*, *P < 0.05, **P < 0.01, ***P < 0.001, ns means no significance.

Previous studies have shown that D_2_-MSNs play an essential regulatory role in pain and negative emotions. To further explore the potential role of D_2_-MSNs in regulating pain and negative emotions in the NAcS, we used chemogenetics to selectively activate D_2_-MSNs and assess their effects on pain and despair-like behaviors. First, we injected hM3Dq (AAV2/9-D_2_-hM3Dq-mCherry) into the NAcS, while control mice received an equivalent volume of control virus (AAV2/9-D_2_-mCherry) (Figures 2A,B). Both experimental and control groups were subjected to identical CNO administration protocols to control for potential off-target effects. *Ex vivo* whole-cell recordings in acute NAcS slices showed that CNO perfusion increased neuronal excitability in hM3Dq-mCherry-expressing NAcS neurons (Figure 2C). Von Frey and Hargreaves behavioral tests indicated that a single intraperitoneal dose of CNO (3 mg/kg, i.p., administered 30 min before testing) sufficiently reduced 50%PWTs and PWL, whereas no significant changes were observed in control virus-injected mice receiving the same CNO treatment, with no effect on FST immobility time (Figures 2D–F). In contrast, repeated CNO administration for 7 days (3 mg/kg, i.p., once daily) not only reduced 50%PWTs and PWL in mice injected with hM3Dq but also increased FST immobility time compared to mice receiving control virus, while control virus-injected mice receiving repeated CNO did not exhibit these behavioral alterations (Figures 2G,H). These results indicate that chemogenetic activation of NAcS D_2_-MSNs is sufficient to induce real-time hyperalgesia, whereas the emergence of despair-like behavior requires prolonged/repeated activation of NAcS D_2_-MSNs.

**FIGURE 3:**
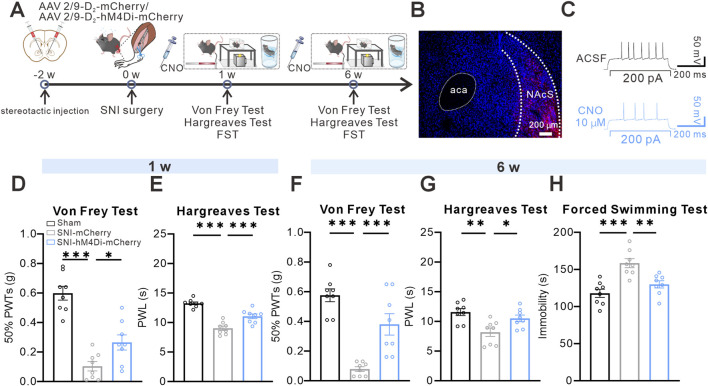
Chemogenetic inhibition of NAcS D_2_-MSNs alleviated SNI-induced pain-depression comorbidity **(A)** Timeline of the SNI surgery procedure and experimental protocol **(B)** Representative confocal images for virus expression in NAcS, scale bar = 200 µm **(C)** Sample trace of whole-cell recording showing the excitatory effect of CNO on an hM4Di-mCherry-expressing NAcS neuron **(D,E)** 50% PWTs **(D)** and PWL **(E)** 30 min after a single CNO administration 1 W following SNI surgery (n = 8 mice/group) **(F–H)** 50% PWTs **(F)**, PWL **(G)**, and immobile time in the FST **(H)** 30 min after a single CNO administration 6 W following SNI surgery (n = 8 mice/group). Data are represented as *mean ± s.e.m.*, *P < 0.05, **P < 0.01, ***P < 0.001.

To assess the necessity of activating D_2_-MSNs in PDC, we established a neuropathic pain-related SNI mouse model (Figure 3A). We replaced AAV2/9-D_2_-hM3Dq-mCherry with AAV2/9-D_2_-hM4Di-mCherry, with SNI-control mice receiving AAV2/9-D_2_-mCherry, and performed the same chemogenetic viral surgery (Figures 3A–C). Both the hM4Di and SNI-control groups received identical CNO administration protocols. One week after SNI surgery, behavioral tests using von Frey and Hargreaves showed that a single dose of CNO (3 mg/kg, i.p., administered 30 min before testing) increased the 50%PWTs and PWL in SNI mice injected with AAV2/9-D_2_-hM4Di-mCherry, whereas no significant effects were observed in control virus-injected SNI mice receiving CNO (Figures 3D,E). Similarly, 6 weeks after SNI surgery, acute CNO administration (3 mg/kg, i.p., administered 30 min before testing) increased the 50%PWTs and PWL in SNI mice injected with AAV2/9-D_2_-hM4Di-mCherry (Figures 3F,G) and decreased immobility time in the FST (Figure 3H), indicating that chemogenetic inhibition of NAcS D_2_-MSNs in SNI-PDC mice also exhibited antidepressant effects in the FST. These findings strongly suggest that inhibiting NAcS D_2_-MSNs alleviates neuropathic pain behavior and attenuates despair-like behavior in SNI-PDC mice.

### Cell type-specific expression of A_2A_Rs in the NAcS

**FIGURE 4:**
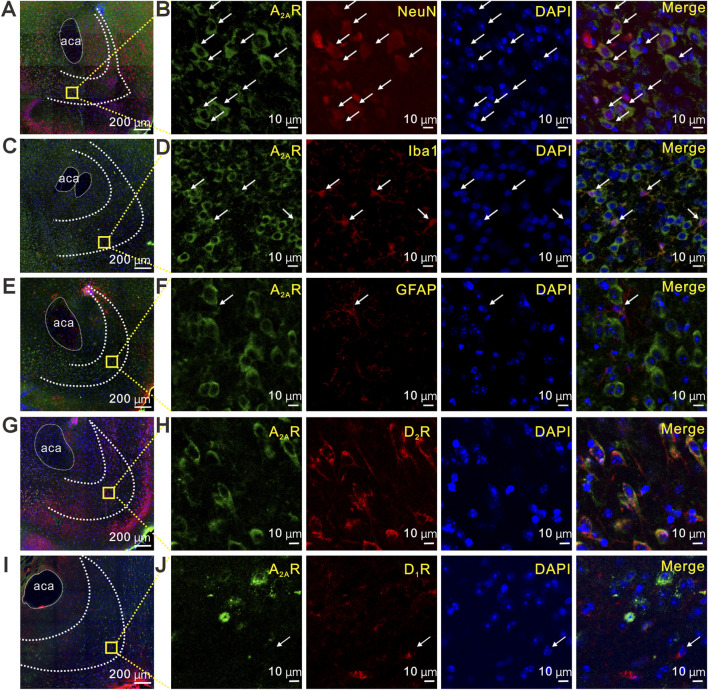
Cell type-specific expression of A_2A_Rs in the NAcS **(A,B)** Representative immunofluorescent images showing the expression of A_2A_Rs (green) and staining of NeuN for neurons (red) **(C,D)** Representative immunofluorescent images showing the expression of A_2A_Rs (green) and staining of Iba1 for microglia (red) **(E,F)** Representative immunofluorescent images showing the expression of A_2A_Rs (green) and staining of GFAP for astrocytes (red) **(G,H)** Representative immunofluorescent images showing the expression of A_2A_Rs (green) and D_2_Rs (red) **(I,J)** Representative immunofluorescent images showing the expression of A_2A_Rs (green) and D_1_Rs (red). Scale bar = 200 µm in **(A,C,E,G,I)**, scale bar = 10 µm in **(B,D,F,H,J)**.

To characterize the cellular localization of A_2A_Rs in the NAcS, we performed immunofluorescence co-staining with the neuronal marker Neuronal Nuclei (NeuN). A_2A_R immunoreactivity was readily detected in NeuN-positive cells, indicating neuronal expression of A_2A_Rs in the NAcS (Figures 4A,B). Similar immunofluorescent co-staining was performed using the microglial marker ionized calcium-binding adapter molecule 1 (Iba1, Figures 4C,D) and the astrocytic marker glial fibrillary acidic protein (GFAP, Figures 4E,F). A_2A_R signals showed prominent colocalization with Iba1 (Figures 4C,D), whereas little to no overlap was observed with GFAP (Figures 4E,F). These data indicate that A_2A_Rs exhibit cell-type-specific expression in the NAcS, primarily localizing to neurons and microglia rather than astrocytes.

In the NAcS, D_1_ and D_2_ receptors are predominantly distributed (Conrad et al., 2010), both of which play crucial roles in regulating neurocircuitry associated with emotion and pain (Ikemoto et al., 1997; Domingues et al., 2025; Wang et al., 2023). Previous studies have confirmed the widespread expression of A_2A_Rs in the brain and have further revealed their specific distribution in the NAcS (Zhang et al., 2013). Immunofluorescence analysis revealed substantial cellular-level colocalization of A_2A_Rs with D_2_Rs (Figures 4G–J), suggesting that A_2A_Rs are enriched in D_2_R-positive cell populations in the NAcS.

### Pharmacological manipulation of A_2A_Rs bidirectionally modulates D_2_-MSNs excitability in the NAcS

**FIGURE 5:**
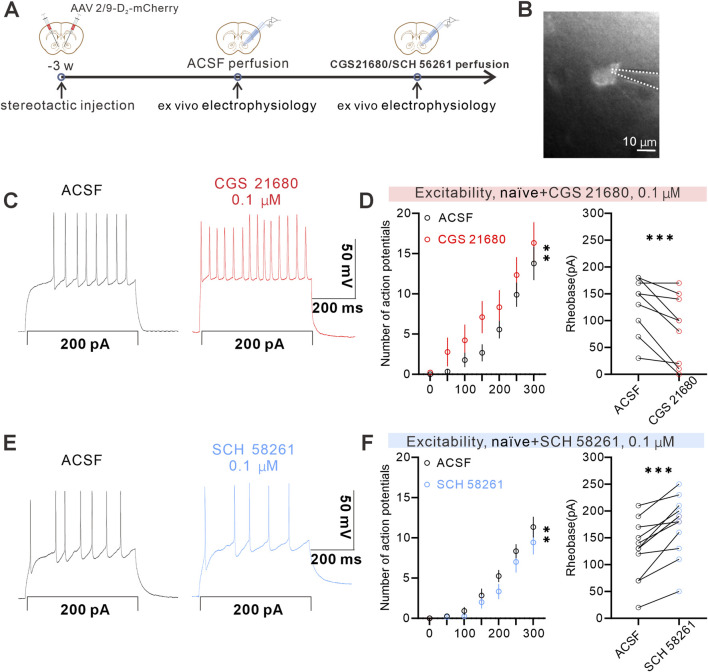
A_2A_Rs modulated the excitability of D_2_-MSNs in the NAcS **(A)** Timeline of the stereotactic injection and *ex vivo* electrophysiology **(B)** Typical micrograph showing the electrophysiological recording of mCherry-labeled NAcS D_2_-MSNs, scale bar = 10 µm **(C)** Sample of whole-cell recording of action potentials in the NAcS after 0.1 µM CGS 21680 perfusion **(D)** (left) 0.1 µM CGS 21680 perfusion increased the number of eAPs of mCherry-labeled neurons (right) The minimal voltage threshold to induce eAPs was lower after perfusion with 0.1 µM CGS 21680 (n = 9 cells from 4 mice) **(E)** Sample of whole-cell recording of action potentials in the NAcS after 0.1 µM SCH 58261 perfusion **(F)** (left) 0.1 µM SCH 58261 perfusion decreased the number of eAPs of mCherry-labeled neurons (right) The minimal voltage threshold to induce eAPs was higher after perfusion with 0.1 µM SCH 58261 (n = 12 cells from 4 mice) Data are shown as *mean ± s.e.m.*, **P < 0.01, ***P < 0.001.

Next, we explored how A_2A_R signaling in the NAcS modulates the excitability of D_2_-MSNs. We injected the AAV2/9-D_2_-mCherry virus into the NAcS (Figures 5A,B) to specifically label D_2_-MSNs. Subsequently, we assessed neuronal excitability by quantifying eAPs. In isolated NAcS slices prepared from naïve mice, perfusion with the A_2A_R agonist CGS 21680 (0.1 μM) significantly increased the number of eAPs in D_2_-MSNs and reduced the rheobase (the minimal current required to evoke an action potential) (Figures 5C,D), indicating an enhancement of intrinsic excitability. To further validate the regulatory role of A_2A_Rs, we tested the selective A_2A_R antagonist SCH 58261 (0.1 μM) under the same conditions. In isolated NAcS slices from naïve mice, perfusion with the A_2A_R antagonist SCH 58261 (0.1 μM) significantly decreased the number of eAPs, and increased the rheobase (Figures 5E,F) in NAcS D_2_-MSNs, showing that the A_2A_R antagonist downregulated NAcS D_2_-MSNs neuronal excitability. Collectively, these electrophysiological data indicate that A_2A_Rs bidirectionally modulate the excitability of D_2_-MSNs in the NAcS, with agonist activation enhancing and antagonist blockade suppressing neuronal activity.

### Pharmacological manipulation of NAcS A_2A_Rs activity bidirectionally modulates pain and despair-like behaviors

**FIGURE 6:**
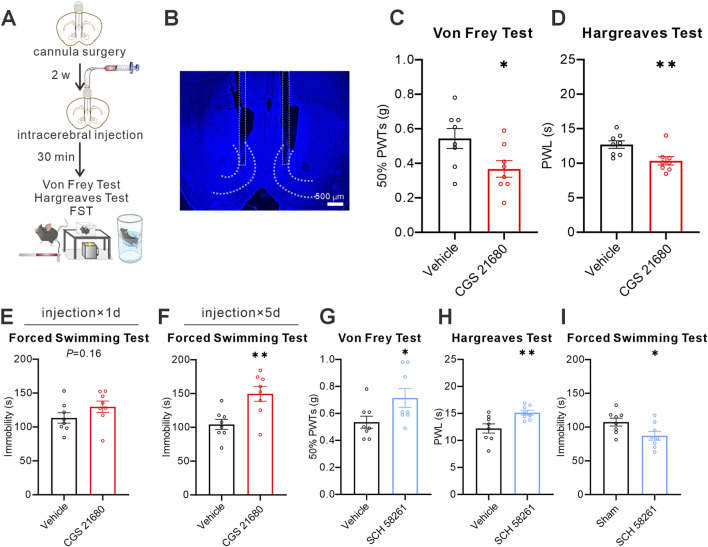
Pharmacological manipulation of NAcS A_2A_Rs regulated pain and depression-like behaviors in naïve mice **(A)** Timeline of the cannula surgery procedure and behavioral tests **(B)** A representative confocal image for cannula placement, scale bar = 500 μm **(C,D)** 50%PWTs **(C)** and PWL **(D)** in mice treated with vehicle or CGS 21680 (2 ng/side) (n = 8 mice/group) **(E,F)** Immobility in FST after single **(E)** or five treatments **(E)** of CGS 21680 (2 ng/side) (n = 8 mice/group) **(G–I)** 50%PWTs **(G)**, PWL **(H)**, and immobility in FST **(I)** in mice after a single treatment with vehicle or SCH58261 (4 ng/side) (n = 8 mice/group). Data are shown as *mean ± s.e.m.*, *P < 0.05, **P < 0.01.

To determine whether local pharmacological manipulation of NAcS A_2A_R activity modulates pain and despair-like behaviors in naïve and SNI mice, we bilaterally implanted guide cannulas targeting the NAcS 2 weeks before behavioral tests for intra-NAcS microinjections of the A_2A_R agonist or antagonist (Figures 6A,B). Our behavioral tests demonstrated that a single microinjection of the A_2A_R agonist CGS 21680 (2 ng/side) significantly decreased the 50%PWTs (Figure 6C) and PWL (Figure 6D) in naïve mice under physiological conditions, with no significant effect on immobility time in the FST (Figure 6E). No significant effect on motor activity was observed in the OPT (Supplementary Figure S1C). However, five times microinjections of CGS 21680 (2 ng/side) increased immobility time in the FST (Figure 6F). We next examined the behavioral consequences of intra-NAcS A_2A_R antagonism using the selective antagonist SCH 58261 (4 ng/side). We found that a single microinjection of SCH 58261 increased 50%PWTs and PWL in naïve mice (Figures 6G,H) and decreased immobility time in the FST (Figure 6I). No significant effect on motor activity was observed in the OPT (Supplementary Figure S1D). These pharmacological findings suggest that A_2A_Rs exert bidirectional regulatory effects on pain and depression-like behaviors in the NAcS.

**FIGURE 7:**
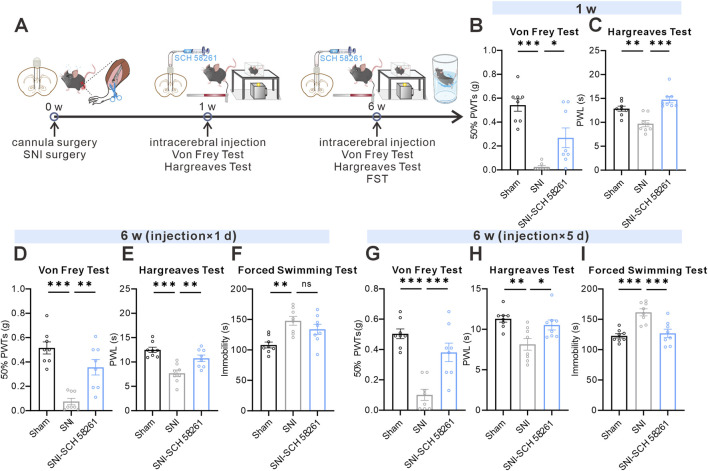
NAcS A_2A_Rs antagonism alleviated SNI-induced pain-depression comorbidity **(A)** Timeline of cannula and SNI surgery, intracerebral injection, Von Frey and Hargreaves tests at specified time points **(B,C)** 50%PWTs **(B)** and PWL **(C)** in mice treated with vehicle or SCH 58261 (4 ng/side) 1 W following SNI surgery (n = 8 mice/group) **(D–F)** 50% PWTs **(D)**, PWL **(E)**, and immobile time in the FST **(F)** after a single microinjection of vehicle or SCH 58261 (4 ng/side) 6 W following SNI surgery (n = 8 mice/group) **(G–I)** 50% PWTs **(G)**, PWL **(H)**, and immobile time in the FST **(I)** after five consecutive microinjections of vehicle or SCH 58261 (4 ng/side) 6 W following SNI surgery (n = 8 mice/group). Data are represented as *mean ± s.e.m.*, *P < 0.05, **P < 0.01, ***P < 0.001, ns means no significance.

To further validate the analgesic effects of NAcS A_2A_R antagonism in SNI comorbid mice, SCH 58261 (4 ng/side) was injected into the NAcS 30 min before behavioral tests (Figure 7A). Compared to the saline-injected SNI group, a single microinjection of the A_2A_R antagonist SCH 58261 (4 ng/side) significantly increased 50%PWTs (Figures 7B,D) and PWL (Figures 7C,E) in mice at week 1 and week 6 after SNI, with no significant effect on immobility time in the FST (Figure 7F). Repeated administration of SCH 58261 for 5 consecutive days (once per day) not only increased the 50%PWTs and PWL (Figures 7G,H) in SNI-PDC mice but also decreased immobility time in the FST (Figure 7I). These results suggest that NAcS A_2A_R antagonism attenuates neuropathic pain during both early and later post-SNI phases, and that chronic treatment is required to produce robust antidepressant-like efficacy in the FST in the established PDC state.

## Discussion

Previous studies have shown that 39.3% of patients with chronic pain also experience depression (Aaron et al., 2025), highlighting the common co-occurrence of depression and chronic pain in individuals with physical illnesses. Traditional monotherapies for either pain or depression often fail to provide synergistic relief for both sets of symptoms. This inadequacy can lead to treatment resistance, relapse, and a growing reliance on polypharmacy, which may contribute to increased opioid consumption and a high risk of adverse effects (Zahlan et al., 2023; Nadeau et al., 2021; Rosoff et al., 2021; Emery and Akil, 2020). NAcS is a critical area in mediating pain and negative emotions (Castro et al., 2019; Mitrano et al., 2012; Xie et al., 2014). Compelling evidence strongly implicates the NAcS as a promising target for developing potential analgesics and antidepressants (Zhao et al., 2024; Yu et al., 2025). However, the cellular and molecular mechanisms by which NAcS mediates this process remain unclear. Using the SNI-induced PDC model, we demonstrate that A_2A_R signaling in the NAcS is associated with PDC through modulation of D_2_-MSN excitability. At the cellular level, A_2A_R agonism increases, whereas A_2A_R antagonism decreases, the excitability of D_2_-MSNs, changes that parallel the direction of behavioral effects. These findings suggest a potential functional role for A_2A_Rs in the NAcS in the context of pain-induced depression comorbidity, and identify A_2A_Rs as a candidate molecular target for potential intervention strategies.

Accumulating evidence indicates that D_2_-MSNs in the NAcS contribute to the regulation of pain and depression. In CCI mice, chemogenetic inhibition of NAcS D_2_-NSNs during the acute phase exhibits analgesic effects, and prolonged inhibition effectively prevents the chronicization of pain (Yu et al., 2025). CCL2/CCR2 signaling in the NAcS relieves pain and depression-like behaviors in SNL mice by downregulating NR2B-mediated NMDAR function in D_2_-MSNs (Wu et al., 2018). Similarly, our results demonstrate that chemogenetic activation of NAcS D_2_-MSNs in naïve mice induces PDC phenotypes. In contrast, chemogenetic inhibition of NAcS D_2_-MSNs in SNI mice effectively alleviates pain and despair-like behaviors in PDC mice, confirming the potential role of D_2_-MSNs in the NAcS in pain and emotion regulation, and further validating D_2_-MSNs as potential targets for treating PDC. Although we found that inhibition of D_2_-MSNs effectively alleviates pain and despair-like behaviors, previous studies have suggested that activation of D_2_-MSNs has an analgesic effect (Soares-Cunha et al., 2020), which contrasts with our results. This discrepancy may be attributed to the following factors: 1) differences in the research regions (such as NAc Shell vs. NAc Core, NAc Medial vs. Lateral) and their distinct neural circuits, which could lead to different downstream effects triggered by D_2_-MSNs activation or inhibition; 2) the acute vs. chronic phases of pain, where D_2_-MSNs may play completely different roles. Therefore, although our current study highlights D_2_-MSNs in the NAcS as potential targets for analgesic and antidepressant therapies, it is essential to consider pain models, the specific neural circuits involved, and other key variables when applying this mechanism.

A series of epidemiological, genetic, and pharmacological studies has highlighted the potential of A_2A_Rs as a novel therapeutic target for pain and depression (El Yacoubi et al., 2001; Jiang et al., 1996; Bura et al., 2008). In a mouse model of trigeminal neuralgia, astrocyte and microglial activation in the ventral hippocampus (vCA1) region cooperatively regulate extracellular adenosine levels and modulate vCA1 pyramidal neuron excitability via A_2A_Rs, thereby mediating anxiety- and depression-like behaviors induced by chronic pain (Lv et al., 2024). Inhibition of astrocyte and microglia activation in the vCA1 reduces the increase in extracellular adenosine levels caused by trigeminal nerve injury and improves the resulting anxiety and depression behaviors (Lv et al., 2024). Chronic stress decreases synaptic plasticity and the density of synaptic proteins (synaptophysin, synapsin, and vesicular glutamate transporter 1) in the hippocampus of mice, while increasing the density of A_2A_Rs in hippocampal glutamatergic terminals (Kaster et al., 2015). Treatment with the A_2A_R antagonist SCH58261 (0.1 mg/kg, intraperitoneal injection) for 3 weeks can reverse the emotional and synaptic dysfunction caused by chronic stress, indicating that synaptic A_2A_Rs play a key role in the changes induced by chronic stress (Kaster et al., 2015). Therefore, identifying the key mechanisms through which A_2A_Rs contribute to analgesic and antidepressant effects remains central to the development of A_2A_R-based therapeutic drugs. It is important to note that A_2A_R modulation can produce divergent effects on nociception depending on anatomical location. In the NAc and PVT, A_2A_R activation has been reported to facilitate pain-related behaviors, whereas local A_2A_R antagonism can attenuate hypersensitivity (Sardi et al., 2018; Cao et al., 2024). In contrast, at the spinal level, A_2A_R agonism has been reported to exert antinociceptive actions (Regay et al., 2004; By et al., 2011). Therefore, the analgesic or pronociceptive outcome of targeting A_2A_Rs likely reflects region- and circuit-specific engagement. This regional variability necessitates careful consideration of the site of action and its impact on the overall neural network when developing therapeutic strategies. Thus, future research aimed at exploring the mechanisms of A_2A_R signaling in different brain regions and the spinal cord will be crucial in better understanding the role of A_2A_Rs in nociception and depression. This knowledge will pave the way for the development of more targeted and effective treatment options.

Moreover, numerous studies suggest that A_2A_Rs and D_2_Rs interact functionally in the NAcS and may form heterodimers (Canals et al., 2003; Kamiya et al., 2003). A_2A_R activation recruits cAMP/PKA signaling to enhance the intrinsic excitability and firing activity of D_2_-MSNs (Wang and Zhou, 2019). In contrast, D_2_Rs suppress cAMP signaling, generally decreasing neuronal excitability and functionally dampening NMDAR-dependent excitatory responses (Azdad et al., 2009). However, the functional interaction between A_2A_Rs and D_2_Rs in the NAcS in the context of PDC remains unclear. This study fills and extends this gap. Our findings demonstrate the critical role of A_2A_Rs in the treatment of PDC. In the NAcS, A_2A_Rs modulate the excitability of D_2_-MSNs, bidirectionally regulating PDC. Pharmacological activation of NAcS A_2A_Rs upregulates the excitability of D_2_-MSNs, inducing PDC in naïve mice. Conversely, pharmacological antagonism of NAcS A_2A_Rs downregulates the excitability of D_2_-MSNs, effectively alleviating pain and despair-like behaviors in SNI mice. Our results suggest significant potential for A_2A_R signaling pathway drugs in treating PDC, providing a new molecular target for the development of future PDC treatment strategies. A_2A_Rs, as a Gs/olf-coupled receptor, upregulate the cAMP/PKA pathway (Chen et al., 2025). A_2A_Rs can inhibit D_2_R signaling by antagonizing the A_2A_R-D_2_R complex at the receptor level (Prasad et al., 2021). A_2A_Rs may also enhance the intrinsic excitability and excitatory synaptic transmission of D_2_-MSNs by modulating ion channels (e.g., Kir2 and voltage-gated Ca^2+^ channels) (Wang and Zhou, 2019; Rendon-Ochoa et al., 2022; Swapna et al., 2016) and glutamate receptor function (e.g., enhancing NMDAR-mediated currents) (Martins et al., 2020). However, the precise signaling pathway by which A_2A_Rs in D_2_-MSNs exert their functional effects remains to be elucidated.

The majority of NAcS neurons are MSNs, which can be classified into D_1_-MSNs expressing dopamine D_1_Rs and D_2_-MSNs expressing dopamine D_2_Rs (Le Moine and Bloch, 1996; Lu et al., 1998; Ikemoto et al., 1997). In this study, we focused on the interaction between A_2A_Rs and D_2_-MSNs in the NAcS and how this interplay bidirectionally modulates PDC. However, the functional segregation or cooperation between D_1_-MSNs and D_2_-MSNs in response to emotional stimuli in the NAcS remains to be elucidated. Previous studies suggest that D_1_Rs and D_2_Rs in the NAc play synergistic (Domingues et al., 2025; Beutler et al., 2011; Perreault et al., 2012) or antagonistic roles (Liang et al., 2022; Sato et al., 2022; Alderete et al., 2025) in regulating pain and negative emotions. Although our current results indicate relatively low co-expression of A_2A_Rs and D_1_Rs, D_1_-MSNs may still play a role in analgesic and antidepressant effects associated with A_2A_ signaling. At baseline dopamine concentrations, D_1_Rs cannot be activated, but D_2_Rs can be activated, thereby inhibiting the activity of D_2_-MSNs. At higher dopamine concentrations, D_1_Rs are activated, which in turn activate D_1_-MSNs while also activating D_2_Rs to inhibit the activity of D_2_-MSNs. At lower dopamine concentrations, neither D_1_Rs nor D_2_Rs are activated, and D_2_-MSNs become active under the inhibitory influence of D_2_Rs. Moreover, basal adenosine continuously activates A_2A_Rs through a homeostatic mechanism. In summary, the synergistic action of dopamine and adenosine plays a switch-like role in regulating the activity switch between D_1_-MSNs and D_2_-MSNs, thereby executing this crucial regulatory function within neural circuits (Zhang et al., 2019). These findings suggest that the activation or antagonism of A_2A_Rs may influence D_1_Rs signaling pathways, thereby indirectly affecting the activity levels of D_1_-MSNs. Therefore, whether NAcS D_1_-MSNs contribute to analgesic and antidepressant effects associated with A_2A_ signaling requires further investigation through cellular and behavioral experiments.

Several limitations of our study warrant consideration. First, we employed only the SNI-induced PDC model. Further validation in other chronic pain-induced depression comorbidity models (such as CCI and inflammatory models) and stress/depression models is necessary to confirm its reproducibility and generalizability. Second, while our data suggest that local modulation of A_2A_R activity in the NAcS can bidirectionally modulate the intrinsic excitability of D_2_-MSNs and pain-associated behavioral despair, the present study was not designed to systematically quantify A_2A_R expression or receptor availability across disease stages. Moreover, although our pharmacological and electrophysiological data support a potential role for A_2A_R signaling, future work employing cell-type-specific knockdown or overexpression approaches will be important to directly test the necessity of A_2A_Rs in D_2_-MSNs for the observed analgesic and antidepressant effects. Additionally, rescue experiments could test whether A_2A_R antagonism loses efficacy when D_2_-MSNs are chemogenetically activated, or conversely, whether inhibition of D_2_-MSNs can block the effects of A_2A_R activation. It will also be of interest to determine whether chronic neuropathic pain or PDC states are accompanied by adaptive changes in A_2A_R expression or signaling strength specifically within NAcS D_2_-MSNs, and how such adaptations relate to the emergence and persistence of affective comorbidities. Third, studies have shown that chronic pain can activate glial cells, resulting in increased extracellular adenosine levels, the activation of A_2A_Rs, and the exacerbation of anxiety and depression-like behaviors (Lv et al., 2024). Glial cells may play a crucial role in regulating pain and negative emotions mediated by the adenosine system. Our immunofluorescence data also reveal that A_2A_Rs are expressed not only in D_2_-MSNs but also in microglia within the NAcS. While our pharmacological manipulations of A_2A_Rs produced behavioral effects consistent with modulation of D_2_-MSN activity, we cannot determine from these experiments whether microglial A_2A_Rs also contribute to these behavioral outcomes. Cell-type-specific manipulations would be required to dissect the relative contributions of neuronal versus microglial A_2A_Rs. Furthermore, our study employed the FST as the primary measure of affective dysfunction in chronic pain. While the FST provides a validated assessment of behavioral despair and passive coping strategies, it captures only a limited dimension of the depression-like phenotype. The NAc serves as a central hub for integrating reward and aversion signals to guide motivated behavior (Zhou et al., 2022). Our study employed only the FST, which measures behavioral despair but does not capture anhedonia, motivational deficits, social withdrawal, or cognitive dysfunction. Future studies should incorporate the sucrose preference test (SPT) to assess anhedonia, social interaction tests (SIT) to evaluate social behavior, operant conditioning to measure motivation, and cognitive tasks to probe decision-making. Such comprehensive assessments would clarify whether D_2_-MSNs’ A_2A_ signaling specifically modulates behavioral despair or more broadly influences multiple dimensions of affective dysfunction in chronic pain.

In conclusion, our study confirms that bidirectional modulation of D_2_-MSNs excitability by A_2A_Rs in the NAcS contributes to both analgesic and antidepressant effects, suggesting a novel mechanism for treating PDC and laying the groundwork for developing potential intervention strategies targeting A_2A_Rs.

## Data Availability

The original contributions presented in the study are included in the article/Supplementary Material, further inquiries can be directed to the corresponding authors.

## Supplementary material

SUPPLEMENTARY FIGURE S1 Effects of CGS 21680 and SCH 58261 on locomotor activity in the open field test. **(A)** Timeline of cannula surgery, intracerebral injection, and open field test at specified time points. **(B)** Total distance traveled in the OFT 30 min after intra-NAcS microinjection of CGS 21680 (25 ng/side) (n = 8 mice/group). **(C)** Total distance traveled in the OFT 30 min after intra-NAcS microinjection of CGS 21680 (2 ng/side) (n = 8 mice/group). **(D)** Total distance traveled in the OFT 30 min after intra-NAcS microinjection of SCH 58261 (4 ng/side) (n = 8 mice/group). Data are represented as *mean ± s.e.m.*, ****P* < 0.001, ns means no significance.
