# Supplementary figures and images for: Adenosine A2A receptors regulate D2-type medium spiny neurons in the nucleus accumbens to mediate pain and depression comorbidity
Source: Front Pharmacol. 2026 Feb 23;17:1759544. doi: 10.3389/fphar.2026.1759544 (PMC12968297; doi:10.3389/fphar.2026.1759544)

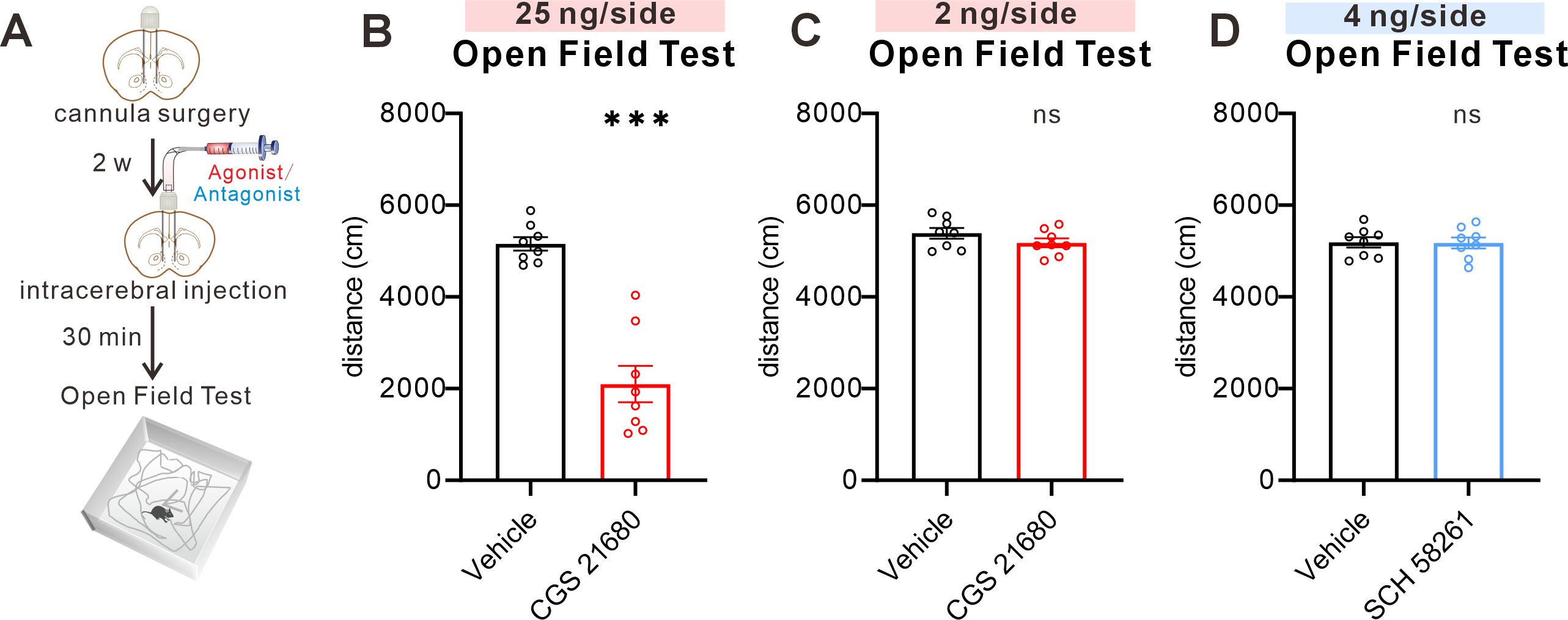

Supplement: Supplementary file 1 [file Image1.tif]
